# Myocarditis and Pericarditis following COVID-19 Vaccination in Thailand

**DOI:** 10.3390/vaccines11040749

**Published:** 2023-03-28

**Authors:** Chayanit Mahasing, Pawinee Doungngern, Rittichai Jaipong, Poonyaporn Nonmuti, Jirapa Chimmanee, Jurai Wongsawat, Thananya Boonyasirinant, Chaisiri Wanlapakorn, Pattranee Leelapatana, Teerapat Yingchoncharoen, Tachapong Ngarmukos, Kulkanya Chokephaibulkit, Suphot Srimahachota

**Affiliations:** 1 Division of Epidemiology, Department of Disease Control, Ministry of Public Health, Building 10 Floor 3, 88/21 Tiwanon Rd., Nonthaburi 11000, Thailand; pawind@gmail.com (P.D.);; 2 Bamrasnaradura Infectious Diseases Institute, Nonthaburi 11000, Thailand; 3 Faculty of Medicine, Siriraj Hospital, Mahidol University, Bangkok 10700, Thailand; 4 Faculty of Medicine, Chulalongkorn University, Bangkok 10330, Thailand; 5 Faculty of Medicine, Ramathibodi Hospital, Mahidol University, Bangkok 10400, Thailand

**Keywords:** adverse events, COVID-19 vaccines, myocarditis, pericarditis, BNT162b2

## Abstract

Background: Myocarditis and pericarditis cases following Coronavirus 2019 (COVID-19) vaccination were reported worldwide. In Thailand, COVID-19 vaccines were approved for emergency use. Adverse event following immunization (AEFI) surveillance has been strengthened to ensure the safety of the vaccines. This study aimed to describe the characteristics of myocarditis and pericarditis, and identify the factors associated with myocarditis and pericarditis following COVID-19 vaccination in Thailand. Method: We carried out a descriptive study of reports of myocarditis and pericarditis to Thailand’s National AEFI Program (AEFI-DDC) between 1 March and 31 December 2021. An unpaired case–control study was conducted to determine the factors associated with myocarditis and pericarditis after the CoronaVac, ChAdOx1-nCoV, BBIBP-CorV, BNT162b2, and mRNA-1273 vaccines. The cases consisted of COVID-19 vaccine recipients who met the definition of confirmed, probable, or suspected cases of myocarditis or pericarditis within 30 days of vaccination. The controls were people who underwent COVID-19 vaccination between 1 March and 31 December 2021, with no adverse reactions documented after vaccination. Results: Among the 31,125 events recorded in the AEFI-DDC after 104.63 million vaccinations, 204 cases of myocarditis and pericarditis were identified. The majority of them were male (69%). The median age was 15 years (interquartile range (IQR): 13–17). The incidence was highest following the BNT162b2 vaccination (0.97 cases per 100,000 doses administered). Ten deaths were reported in this study; no deaths were reported among children who received the mRNA vaccine. Compared with the age-specific incidence of myocarditis and pericarditis in Thailand before the introduction of the COVID-19 vaccination, the incidence of myocarditis and pericarditis after the BNT162b2 vaccine was greater in the 12–17 and 18–20 age groups in both males and females. It was higher after the second dose in 12- to 17-year-olds (2.68 cases per 100,000 doses administered) and highest after the second dose in male 12- to 17-year-olds (4.43 cases per 100,000 doses administered). Young age and a mRNA-based vaccination were associated with myocarditis and pericarditis following administration of the COVID-19 vaccine after multivariate analysis. Conclusions: Myocarditis and pericarditis following vaccination against COVID-19 were uncommon and mild, and were most likely to affect male adolescents. The COVID-19 vaccine offers the recipients enormous benefits. The balance between the risks and advantages of the vaccine and consistent monitoring of AEFI are essential for management of the disease and identification of AEFI.

## 1. Introduction

Acute myocarditis typically results from viral infections such as enterovirus, adenoviruses, parvovirus B19, and human herpesvirus (HHV4 and HHV6). SARS-CoV-2 infection can also indirectly trigger myocarditis. The existing evidence suggests that the mechanisms of SARS-CoV-2 of myocardial injury include myocardial damage by a cytokine storm, respiratory dysfunction, and hypoxemia [1,2,3,4,5]. The estimated global incidence of common viral acute myocarditis was 1–10 cases per 100,000 people per year [6]. Individuals who are susceptible include children, pregnant women, and those who are immunocompromised [7]. Myocarditis can also be caused by autoimmune diseases with a negative viral PCR as well as by drugs and toxins [8,9,10,11,12,13,14,15]. Vaccine-associated myocarditis is rare. The Vaccine Adverse Events Reporting System (VAERS) documented 0.1% of cases corresponding to acute myocarditis from 620,195 reports [16]. According to VAERS in 2011–2015, vaccines that may cause myocarditis include the smallpox, meningococcal, typhoid, Japanese encephalitis, and anthrax vaccines [17,18]. Currently, post-vaccination myocarditis was reported following the coronavirus 2019 (COVID-19) vaccine, particularly those using mRNA-based technology [6].

Post-mRNA-vaccine myocarditis was reported at an estimated rate of 0.3–5.0 cases per 100,000 vaccinated people [19,20,21,22]. The highest incidence was after the second vaccination and mostly among young men and occur within the first week after the vaccination (typically 3–4 days). The development of the symptoms of myocarditis following the COVID-19 vaccine was shorter than in cases who developed myocarditis following a viral infection [23,24,25]. More than 90% of patients will functionally recover completely, with a mortality rate of less than 1% [6]. Fulminant course, ICU-level support, mortality, and transplantation were rare compared with viral myocarditis [25]. However, we found cases of death sporadically reported after mRNA vaccination [24]. There were reported cases of death following the BNT162b2 vaccine in the elderly. All of them had pre-existing cardiovascular conditions [26,27]. There were no confirmed deaths after mRNA-based COVID-19 vaccination among persons younger than 30 years of age without another identifiable cause [24] mRNA-1273 reported a higher rate than BNT162b2, and increasing the interval between doses reduced the risk of myocarditis [28].

In Thailand, COVID-19 vaccines were approved for emergency use and were implemented in the national program in March 2021. The first vaccine was CoronaVac (by Sinovac), followed shortly by ChAdOx1-nCoV (by AstraZeneca) and BBIBP-CorV (by Sinopharm). BNT162b2 (by Pfizer) and mRNA-1273 (by Moderna) were used in late 2021. Homologous and heterologous regimens were used. In children and adolescents, mRNA vaccines were mainly used. To ensure the vaccines’ safety, AEFI surveillance was strengthened countrywide. Hospitals are requested to report any serious AEFI from COVID-19 vaccines, including myocarditis and pericarditis, to the National AEFI Program (AEFI-DDC). In addition, another self-reporting system called “Mo Phrom” has been established to monitor the vaccine administration program as well as to allow individuals who received the vaccine to report their adverse event using a mobile phone application [29].

Because of the increasing use of the mRNA vaccine, particularly in younger children, safety information is crucial to support the vaccine policy. Myocarditis and pericarditis are concerning AEFI events that received high levels of public attention and may affect vaccine hesitancy. This study aimed to describe the characteristics of myocarditis and pericarditis following COVID-19 vaccination in Thailand. Moreover, we aimed to identify the factors associated with myocarditis and pericarditis following COVID-19 vaccination in Thailand.

## 2. Methods

### 2.1. Study Design

We performed a descriptive study to determine the incidence and clinical presentations and outcomes of myocarditis/pericarditis following COVID-19 vaccination reported in the AEFI-DDC in Thailand. The study period was from 1 March 2021, when COVID-19 vaccination was first implemented in Thailand, to 31 December 2021. Furthermore, an unmatched case–control study was conducted to identify the factors associated with myocarditis/pericarditis following COVID-19 vaccination. the cases were those who received the COVID-19 vaccine and met the definition of confirmed, probable, or suspected cases of myocarditis/pericarditis within 30 days after vaccination. In the analytical study, we excluded cases who had no report of the onset date. The controls were those who received the COVID-19 vaccination from 1 March to 31 December 2021 but had no adverse reaction reported in the AEFI-DDC and Mor Phrom systems within 30 days following vaccination. We sampled controls from the MOPH-IC database, which reported the history of COVID-19 vaccine administration in individuals receiving the COVID-19 vaccines across the country. The controls were sampled using sample random sampling. We included all suspected, probable, and confirmed cases in the descriptive and analytical studies.

### 2.2. Data Extraction and Management

We extracted the data from 4 databases, including the AEFI-DDC, the COVID-19 case report database, the MOPH-IC database, and the 43-Files database. The AEFI-DDC’s reporting system is the well-established passive reporting system of serious AEFI including death, life-threatening events, permanent handicaps, congenital disabilities, or hospital admission >3 days [29]. This reporting system links to all hospitals under the MOPH and is responsible for monitoring the vaccine safety of the National Immunization Program. It has been set up for more than 20 years, and has been recently updated from a paper-based fax system to an online system. The system was prepared for the COVID-19 national vaccination program by enhancing the workflow system, acquiring more staff, and providing repeat training. The COVID-19 case report database reports the history of COVID-19 infections. This database system was newly set up in response to the outbreak. It is reported by the Provincial Health Office to the national level. COVID-19 has been included the Communicable Diseases Act B.E. 2558, by which infections have to be reported by law. The MOPH-IC Database reports the vaccine history, including type, dose, and date administered. As all the COVID-19 vaccines were used under the emergency utilization approval (EUA), the vaccination data were reported into the national online system by the vaccination center. The system was linked to individuals’ ID and the vaccination history report available on the mobile telephone application called “Mor Phrom” for each person, which also generated vaccine certificates. Moreover, the Mor Phrom system also allowed the vaccinees to self-report any adverse events following vaccination. Lastly, the 43-Files database reports the annual number of myocarditis/pericarditis cases in Thailand. It includes reports by hospitals with the ICD-10 code I40-I41.8. This report system is linked to the Universal Coverage system and re-imbursement is given to the hospital by the MOPH.

We merged the COVID-19 infection histories from the COVID-19 case report database and the vaccine administration histories from the MOPH-IC database using each person’s 13-digit national identification number. We reviewed the medical records of myocarditis/pericarditis cases to explore information about the clinical and laboratory investigations. We calculated the median incidence of myocarditis/pericarditis from the 43-Files database during 2018–2020, prior to the COVID-19 outbreak, to compare this with the study period after implementation of the COVID-19 vaccination program.

### 2.3. Study Definition

We classified myocarditis/pericarditis into suspected, probable, and confirmed cases. Confirmed and probable cases were based on the definitions of the US-CDC criteria [30]. A confirmed myocarditis case was one with presence of at least 1 of the clinical symptoms (chest pain, dyspnea, palpitation, or syncope) and the presence of at least 1 of a histopathologic confirmation or an MRI finding consistent with myocarditis in presence of an abnormal troponin level. The confirmed pericarditis cases were those with the presence of at least two of clinical features of acute chest pain, pericardial rub upon examination, new ST elevation or PR depression on EKG, new or worsening pericardial effusion on echocardiography or MRI examination. The confirmed myopericarditis cases were those that meet the criteria for both myocarditis and pericarditis. Probable cases of myocarditis were those with the presence of at least 1 clinical symptom (chest pain, dyspnea, palpitation, or syncope) and the presence of at least 1 laboratory finding (troponin level above the normal limit, abnormal EKG, abnormal cardiac function on echocardiography, or an MRI finding consistent with myocarditis). Suspected cases of myocarditis, pericarditis, and myopericarditis were defined by a provisional diagnosis at the hospital as reported in the AEFI-DDC database. Since different vaccines were introduced at different time points, we also categorized the time of vaccination into three categories: March–May, June–September, and October to December.

### 2.4. Data Analysis

Both descriptive statistics and inferential statistics were applied using STATA version 14. For the descriptive statistics, we used the frequency and percentage to describe the characteristics and possible risk factors. For inferential statistics, we performed logistic regression to identify the factors associated with myocarditis/pericarditis following the COVID-19 vaccine. The outcome of interest was those who met the definitions of suspected, probable, or confirmed cases. Moreover, another model using those who met the definitions of probable and confirmed case was also performed to ensure the direction of association (Supplementary Table S3). Variables with a *p*-value of <0.20 in the univariate analysis were included in the multivariate analysis; we used the backward elimination procedure to fit the model. Variables with a *p*-value of <0.05 were considered statistically significant. We also compared the incidence of myocarditis/pericarditis following COVID-19 vaccination with the background incidence of myocarditis/pericarditis in 2018–2020 by calculating the 3-year median of myocarditis/pericarditis cases from 43-Files, stratified by sex and age group.

## 3. Results

### 3.1. Characteristics of Myocarditis/Pericarditis following COVID-19 Vaccination

**Figure 1:**
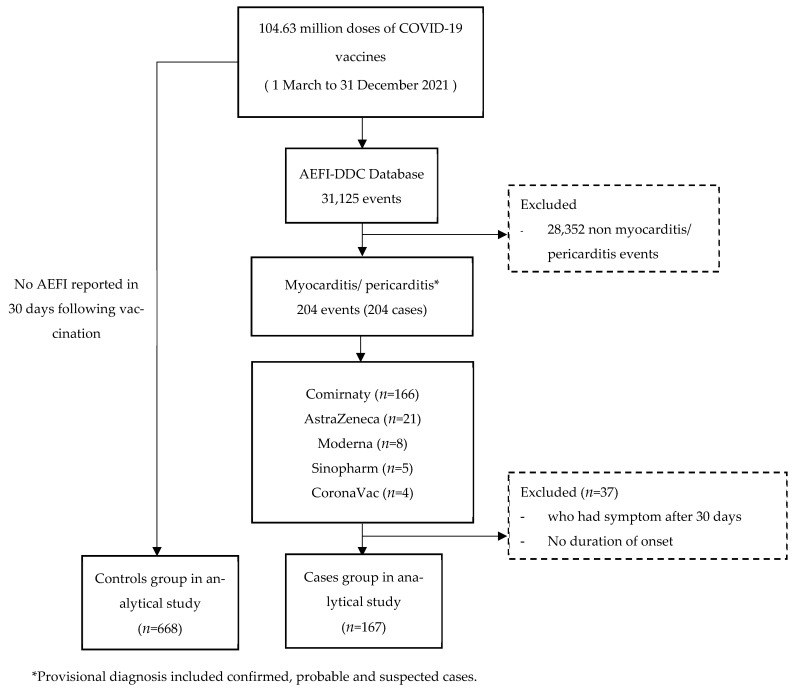
Flow chart of the study.

Of the 31,125 events reported in the AEFI-DDC following 104.63 million vaccinations, 204 cases reported myocarditis/pericarditis (1.95 cases per 1 million doses). Of these, only 71 (35%) cases had their medical records available for review. Of these 204, 153 were suspected cases (75%), 28 (14%) were probable cases, and 23 (11%) fitted the definition of confirmed cases (see more characteristics of the myocarditis, pericarditis, and myopericarditis cases in Supplementary Table S1). Most of them were male (69%). The median age was 15 years (interquartile range (IQR): 13–17). The majority of cases were reported following BNT162b2 (166; 81%), followed by ChAdOx1 nCoV (21; 10%), mRNA-1273 (8; 4%), BBIBP-CorV (5; 3%), and CoronaVac (4; 2%) (Figure 1). By the time of this review, 145 (70%) of the cases had improved or had recovered from their symptoms, but 10 were reported to have died, including nine deaths following ChAdOx1-nCoV (90%) and 1 death following CoronaVac. There were 49 (24%) cases for which the outcome data were not available for this analysis. There were four cases reported as having a history of COVID-19 infection prior to the incidental vaccination (Table 1) (see more characteristics found in the medical record review in Supplementary Table S2).

### 3.2. The Incidence of Myocarditis/Pericarditis Stratified by Age Group, Sex, and Vaccine Type

The highest overall incidence of myocarditis/pericarditis was reported for BNT162b2 (0.97 cases per 100,000 doses administered), followed by MRNA-1273 (0.34 cases per 100,000 doses administered), ChAdOx1-nCoV (0.048 cases per 100,000 doses administered), BBIBP-CorV (0.034 cases per 100,000 doses administered), and CoronaVac (0.015 cases per 100,000 doses administered). (Table 1) As of December 2021, only BNT162b2 and BBIBP-CorV were approved to use in children aged 5–11 and 12–17 years. The only case of suspected myocarditis after the ChAdOx1-nCoV vaccine was a male aged 17 years and 8 months male who was willing to receive the ChAdOx1-nCoV vaccine. In addition, two females, aged 10 and 13 years old, were reported as suspicious myocarditis cases following the BBIBP-CorV vaccine. All of them had fully recovered. BNT162b2, the vaccine recommended for children and adolescents, had a high incidence of myocarditis in males aged 12–17 years (2.87 cases per 100,000 doses administered), females aged 12–17 years (1.03 cases per 100,000 doses administered), and males aged 18–20 years (1.03 cases per 100,000 doses administered) (Table 2). When compared with the background incidence of myocarditis reported before the COVID-19 pandemic, the incidence of myocarditis/pericarditis was six times higher among males aged 12–17 years.

### 3.3. The Incidence of Myocarditis/Pericarditis for BNT162b2, Stratified by Age Group, Sex, and Dose

As the incidence of myocarditis/pericarditis following BNT162b2 was higher than for other types of vaccines, we stratified the incidence of myocarditis/pericarditis reported for BNT162b2 by age, sex, and dose. Compared with the age-specific background incidence of myocarditis/pericarditis in Thailand before implementation of the COVID-19 vaccine, the incidence of myocarditis/pericarditis following BNT162b2 was higher in those aged 12–17 years and 18–20 years for both males and females (Table 2). It was higher after the second dose in those aged 12–17 years (2.68 cases per 100,000 doses administered) and was highest, in males aged 12–17 years, particularly after the second dose (4.43 cases per 100,000 doses administered) (Table 3).

### 3.4. Factors Associated with Myocarditis/Pericarditis following COVID-19 Vaccination in Thailand

Factors associated with myocarditis/pericarditis following the COVID-19 vaccine in Thailand were a young age and mRNA-based vaccines. The univariate analysis showed that the odds of myocarditis/pericarditis for those aged 12–17 years and 18–20 years, male, and receiving an mRNA-based vaccine (BNT162b2 and MRNA-1273) were significantly different compared with those age > 60 years, female, and receiving CoronaVac, respectively. A history of COVID-19 infection was not associated with post-vaccination myocarditis/pericarditis in the univariate analysis (Table 4). In the multivariate analysis, the odds of myocarditis/pericarditis in those aged 12–17 years and 18–20 years were 24 times (aOR = 23.89 (95% CI = 7.20–79.30)) and 5 times (aOR = 4.82 (95% CI = 1.42–16.32)) higher than in those aged >60 years after adjusting for vaccine type and sex. We also found that after adjusting for age and sex, the odds of myocarditis/pericarditis among those who received mRNA-based vaccines BNT162b2 and MRNA-1273 were 13 (aOR = 12.78 (95% CI = 3.05–53.48)) and 66 (aOR = 66.08 [95% CI = 9.81–445.07)) times the odds of CoronaVac, respectively. However, ChAdOx1-nCoV (aOR = 2.26 (95% CI = 0.61–8.40)) did not show a significant difference. Being male was associated with myocarditis/pericarditis in the univariate analysis. However, sex did not significantly differ after controlling for age and the type of vaccine in the multivariate model (Table 5). A similar pattern of association was demonstrated when using probable and confirmed myocarditis cases (see the analytical model of confirmed and probable cases in Supplementary Table S3).

## 4. Discussion

Using the AEFI reporting system of MOPH, covering over 100 million doses of COVID-19 vaccination nationwide, this study described the incidence and characteristics of myocarditis/pericarditis following COVID-19 vaccination in Thailand. Most of the reported cases of myocarditis/pericarditis were in males age 12–17 years, and mRNA vaccines were the most common vaccines involved, consistent with the results previously reported in other settings [20,22,31,32,33]. The incidence was six times higher than the background rate in males aged 12–17 years. As this age group was predominately vaccinated with BNT162b2 under the national program, the incidence rate was higher following BNT162b2 than following mRNA-1273. We found most of the cases recovered or improved without requiring a lengthy hospital stay. Some studies have reported myocarditis deaths following the COVID-19 vaccination. A study in Germany from 27 December 2020 to 12 March 2021 reported five deaths from myocarditis, with their onset ranging from 1 to 50 days after vaccination with BNT162b2. However, all of them had pre-existing cardiovascular diseases that were potential causes of death [26]. Another study in Germany investigated the postmortem findings of 18 fatalities following COVID-19 vaccines. One of them was a 65-year-old male who reported myocarditis after BNT162b2. Moreover, the autopsy found myocarditis in the presence of severe pre-existing cardiac changes [27].

The pathogenesis of vaccine-induced myocarditis is not well understood. A possible mechanism could be the hypersensitivity reaction, the host genetics (e.g., the variants in the genes encoding HLA), immune cross-reactivity, and sex-related factors [6]. The hypotheses most often addressed were the hyperimmune or inflammatory response, autoimmunity triggered by molecular mimicry, delayed hypersensitivity, eosinophilic myocarditis, and hypersensitivity to the vehicle components of the vaccine [34,35,36,37]. As the mRNA vaccine platform is a new technology associated with most cases of myocarditis/pericarditis, we have yet to understand the role of the vaccine’s properties in the mechanism of myocarditis. It is also possible that a combination of factors interplayed with the event. Moreover, vaccine-induced inflammation may trigger or worsen underlying cardiac conditions and result in myocarditis/pericarditis. Vaccination-related stress responses may result in cardiac symptoms, resulting in a misleading diagnosis of myocarditis/pericarditis [38]. It is crucial to understand the characteristics and associated factors of the events in order to prevent and prepare for mass vaccination to control COVID-19 outbreaks.

While inactivated vaccines were introduced widely earlier in the mass vaccination program, we found that myocarditis/pericarditis was rarely reported. Likewise, the ChAdOx1-nCoV vaccine was not found to be of concern. This is in concordance with the report from the UK of an incidence of myocarditis of 3.7 per million doses of ChAdOx1 nCoV, while it was 5.0 per million doses for mRNA vaccines [39,40]. On the contrary, the European Medicines Agency reported the incidence of reported myocarditis to be 2.0 per million doses for viral vector vaccines and 1.6 per million doses for mRNA vaccines [40,41]. This discrepancy is not well explained and underscores the need for further studies.

The incidence of vaccine-induced myocarditis/pericarditis in Thailand is relatively low compared with other studies; however, it was clearly higher than the background rates in adolescents and young adults aged 12–20 years. In this regard, the background incidence of myocarditis/pericarditis might be underestimated, as the database we used mostly covered public hospitals, which account for approximately 72% of the hospitals in the country [42,43]. In the USA, the rates of myocarditis were also highest after the second vaccination dose in adolescent males [21,44,45,46]. The report from VARS showed 7.07, 10.59, and 5.24 myocarditis/pericarditis cases per 100,000 doses after the second dose in males aged 12–15, 16–17, and 18–24 years, respectively [24]. Compared with the USA, reports of myocarditis/pericarditis in Thailand in these risk groups were quite lower than that those reported to VARS [24].

Thailand’s incidence was also lower than that in countries where a greater proportion of adolescents had been vaccinated, with incidence rates of 4.4 to 16.2 per 100,000 vaccinations [47]. The possible reasons might be related to the vaccine recommendations during the study period [48,49,50,51]. Throughout 2021, the policy for vaccine recommendations for children were constantly adjusted for the risk of myocarditis in boys. For example, BNT162b2 was limited to children aged 12–17 years with comorbidities from August to September [49]. Then, in late September to December, according to the precautionary measures resulting from vaccine-induced myocarditis in children, Thailand recommended that healthy boys aged 12–17 years should receive a single dose [50]. The second dose was later introduced with an inadvertently wider interval, which could have resulted in a lower rate of myocarditis [52,53]. Moreover, mRNA was used as the mix regimen in adults and children in the primary series as well; therefore, the higher incidence that was likely to be found for the second homologous mRNA vaccine dose [24,54] may be less apparent. However, our analytical study suggested no statistically significant difference between the first and the third doses of the mRNA vaccine. The inconsistency might be related to the recommendation of a mixed regimen in Thailand [55,56].

Myocarditis/pericarditis was strongly related to adolescents aged 12–17 years. Previous studies also suggested the higher incidence of myocarditis in adolescents, ranging from 1.6 to 37 cases per 100,000 vaccinated persons [20,22,31,32,33,57]. While the incidence of myocarditis/pericarditis in adolescent was higher, the severity was less compared with the older age group. This could be because of the higher frequency of underlying cardiac conditions in those from older age groups. There were no deaths reported for young people who received the mRNA vaccine in this study. The precautionary advice to avoid vigorous exercise may be warranted in male adolescents. The European Society of Cardiology (ESC) [15] suggested that individuals with myocarditis should avoid moderate to high-intensity activity for three to six months [58].

Even though there was a higher incidence of myocarditis and pericarditis among young people, the Advisory Committee on Immunization Practices (ACIP) of the CDC still recommends the mRNA vaccine and concludes that the benefits of the vaccine in terms of preventing morbidity and mortality outweigh the risks of myocarditis after vaccination [30]. Since October 2022, the recent policy has recommended the bivalent Pfizer-BioNTech and Moderna mRNA vaccines as a booster dose for children aged 5 to 11 years in the USA. As of 1 January 2023, there have been no reports of myocarditis or deaths following bivalent booster immunization [59]. On the other hand, SARS-CoV2 infections can indirectly trigger myocarditis through cytokine-mediated cardiotoxicity or by eliciting an autoimmune response against the components of the heart [60]. The incidence of post-vaccine myocarditis was low compared with that of COVID-19 infection-associated myocarditis, which was was 1000–4000 per 100,000 people infected with SARS-CoV-2 [6], even in younger age groups. According to data from 40 health care institutions in the USA participating in a global network, both males and females of all ages had a much greater risk of cardiac problems following SARS-CoV-2 infection than following mRNA COVID-19 immunization. Teenage boys aged 12–17 years had a 2–6 times higher and young men aged 18–29 years had a 7–8 times higher risk of heart complications after infection compared with vaccination [61]. Consistent studies on the risk of myocarditis from infection and vaccination were also reported in Israel [62] and the UK [63]. Our findings support the safety of vaccination despite the increased risk of myocarditis/pericarditis.

This study had some limitations. Firstly, selection bias may have occurred, as the controls were selected from those who reported no AEFI symptoms 30 days after vaccination. Not all vaccinees reported their symptom in the surveillance system. There may be some cases of mild myocarditis missing from the reporting system. This could also have led to a lower incidence rate. Moreover, because of the limits of our database, we could not link with the hospital database to examine the pre-vaccination status of the controls, such as a pre-existing medical condition or cardiovascular risk factor; therefore, a temporal bias is plausible. Secondly, we included all suspected, probable, and confirmed cases in the analysis. There might be some misclassification of cases, as we were unable to confirm the diagnosis in all cases. The medical records were available for review in only one-third of cases. In addition, younger age groups and those who received mRNA vaccines might be more likely to be diagnosed with myocarditis. Thereby, misclassification of the outcomes would be possible. However, we found the same direction of association when performing the analytical study to include only the confirmed and probable cases, suggesting the low chance of overdiagnosis (see Supplementary Table S3). Thirdly, this study demonstrated the situation of a 10-month period following a COVID-19 vaccine campaign in Thailand, with different types of vaccine being introduced at different time points. mRNA Vaccines were introduced later during the campaign. To take the timing of vaccination into account, we included the time period in the analysis. The results showed a similar pattern. Sparse data bias was also possible, since mRNA-1237 had been introduced in November, so a small number of cases and vaccines had been observed. Lastly, the incidence of myocarditis after COVID-19 in this study could have been underestimated because of under-reporting and misdiagnoses with other cardiological symptoms.

## 5. Conclusions

Myocarditis/pericarditis following COVID-19 vaccination were uncommon and were likely to occur in male adolescents. The incidence was higher for the mRNA vaccines than for other types of vaccine. However, the illness was mostly mild, and the incidence in Thailand was low compared with the international data. The COVID-19 vaccine provides tremendous benefits to the recipients, and the incidence of cardiac complications was higher after a SARS-CoV2 infection than after vaccination. Balancing the risks and benefits of the vaccine and continuous monitoring of AEFI are crucial for disease control and the detection of AEFI.

## Figures and Tables

**Table 1:** Characteristics of the myocarditis/pericarditis cases following immunization in Thailand.

	ChAdOx1-nCoV *n* = 21	BNT162b2 *n* = 166	BBIBP-CorV *n* = 5	CoronaVac *n* = 4	MRNA-1273 *n* = 8	Total *n* = 204
No. of case reports to AEFI-DDC	21	166	5	4	8	204
No. of vaccination doses administered	44,159,927	17,137,233	14,578,943	26,385,393	2,371,390	104,632,886
Rate (cases/1 million dose)	0.048	0.970	0.034	0.015	0.337	1.95
Reported age, No.	21	166	5	4	8	202
Age, median (IQR), y	61 (41–66)	14 (13–16)	33 (13–47)	23 (21.5–27.5)	39 (32.5–52)	15 (13–17)
Reported onset, No.	20	163	3	3	8	197
Time to symptom onset, median (IQR), d	4 (1–23)	2 (1–4)	5 (0–44)	3 (2–16)	2 (0–6)	2 (1–4)
Reported sex, No.	21	166	5	4	8	204
Male (%)	13 (61.9)	120 (72.29)	2 (40)	2 (50)	3 (37.5)	140 (68.63)
Female (%)	8 (38.1)	46 (27.71)	3 (60)	2 (50)	5 (62.5)	64 (31.37)
Reported occupation No.	21	166	5	4	8	204
Occupation (%)						
Student	3 (14.29)	135 (81.33)	2 (40)	1 (25)	0 (0)	141 (85.45)
HCP	0 (0)	2 (1.2)	0 (0)	0 (0)	0 (0)	2 (1.21)
Monk	0 (0)	2 (1.2)	0 (0)	0 (0)	0 (0)	2 (1.21)
Government officer	0 (0)	1 (0.6)	0 (0)	1 (25)	0 (0)	2 (1.21)
Merchant	1 (4.76)	1 (0.6)	0 (0)	1 (25)	1 (12.5)	4 (2.42)
Unemployed	3 (14.29)	0 (0)	0 (0)	0 (0)	0 (0)	3 (1.82)
Housework	2 (9.52)	0 (0)	0 (0)	0 (0)	0 (0)	2 (1.21)
Employee	4 (19.05)	0 (0)	1 (20)	0 (0)	2 (25)	7 (4.24)
Farmer	1 (4.76)	0 (0)	0 (0)	0 (0)	1 (12.5)	2 (1.21)
Unknown	7 (33.33)	25 (15.06)	2 (40)	1 (25)	4 (50)	39 (19.12)
Reported history of COVID-19 infection, No.	21	166	5	4	8	204
No	20 (95.24)	164 (98.8)	5 (100)	4 (100)	7 (87.5)	200 (98.04)
Yes	1 (4.76)	2 (1.2)	0 (0)	0 (0)	1 (12.5)	4 (1.96)
Reported dose, No.	21	166	5	4	8	204
Dose 1	5 (23.81)	54 (32.53)	3 (60)	4 (100)	2 (25)	68 (33.33)
Dose 2	16 (76.19)	111 (66.87)	2 (40)	0 (0)	2 (25)	129 (63.24)
Dose 3	0 (0)	1 (0.6)	0 (0)	0 (0)	4 (50)	7 (3.43)
Reported prognosis, No.	21	166	5	4	8	204
Status ^$^ (%)						
Full recovery	1 (4.76)	49 (29.52)	1 (20)	0 (0)	1 (12.5)	52 (25.49)
Improved	6 (28.57)	82 (49.4)	0 (0)	1 (25)	4 (50)	93 (45.59)
Death	9 (42.86)	0 (0)	0 (0)	1 (25)	0 (0)	10 (4.90)
Unknown	5 (23.81)	35 (21.08)	4 (80)	2 (50)	3 (37.5)	49 (24.02)
Reported hospitalization, No.	21	166	5	4	8	204
Hospitalization (%)						
IPD	11 (52.38)	139 (83.73)	2 (40)	1 (25)	7 (87.5)	160 (78.43)
OPD	6 (28.57)	18 (10.84)	1 (20)	2 (50)	1 (12.5)	28 (13.73)
ER	2 (9.52)	7 (4.22)	0 (0)	1 (25)	0 (0)	10 (4.90)
Unknown	2 (9.52)	2 (1.2)	2 (40)	0(0)	0 (0)	6 (2.94)
Reported hospital stay, No.	13	93	2	2	4	114
Duration of hospital stay, median (IQR), in days	3 (1–12)	3 (1–4)	22.5 (10–35)	21 (7–35)	2.5 (1–4.5)	3 (1–5)

IQR, interquartile range; HCP: healthcare provider; ^$^: status was evaluated at date reported. IPD: inpatient department, OPD: outpatient department, ER: emergency room.

**Table 2:** Rate of suspected myocarditis/pericarditis (per 100,000 doses administered) by vaccine, age, and sex (*n =* 202).

Age Group	Vaccine and Sex													
	ChAdOx1-nCoV		BNT162b2		BBIBP-CorV		CoronaVac		MRNA-1273		Total		Background Incidence ^$^ (Cases/100,000 Population)	
	Male	Female	Male	Female	Male	Female	Male	Female	Male	Female	Male	Female	Male	Female
05–11 y	NA	NA	0.00	0.00	0.00	6.21	NA	NA	0.00	0.00	0.00	4.87	0.24	0.29
12–17 y	12.78	0.00	2.87	1.03	2.90	0.00	0.00	0.00	0.00	0.00	2.89	1.02	0.45	0.37
18–20 y	0.14	0.14	1.03	0.47	0.00	0.00	0.00	0.00	0.00	0.00	0.32	0.18	0.87	0.44
21–40 y	0.01	0.01	0.13	0.05	0.00	0.03	0.04	0.04	0.20	0.61	0.03	0.05	0.83	0.51
41–60 y	0.03	0.02	0.07	0.00	0.04	0.04	0.00	0.00	0.57	0.00	0.04	0.02	1.58	1.08
61–80 y	0.19	0.04	0.15	0.00	0.00	0.00	0.00	0.00	0.00	0.61	0.13	0.03	3.88	3.25
>80 y	0.00	0.20	0.00	0.00	0.00	0.00	0.00	0.00	0.00	0.00	0.00	0.11	4.77	4.94

^$^: The background incidence is the 3-year median incidence of myocarditis/pericarditis in 2018–2020 prior to implementation of the COVID-19 vaccine.

**Table 3:** Number of myocarditis/pericarditis cases (per 100,000 doses administered) for the BNT162b2 vaccine, stratified by age, sex, and dose number (*n =* 165).

Age Group	All			Male			Female		
	Dose 1	Dose 2	Dose 3	Dose 1	Dose 2	Dose 3	Dose 1	Dose 2	Dose 3
05–11 y	0.00	0.00	0.00	0.00	0.00	0.00	0.00	0.00	0.00
12–17 y	1.11	2.86	0.00	1.61	4.43	0.00	0.62	1.48	0.00
18–20 y	0.90	0.68	0.00	1.11	1.07	0.00	0.70	0.32	0.00
21–40 y	0.11	0.08	0.08	0.00	0.18	0.20	0.22	0.00	0.00
41–60 y	0.12	0.00	0.00	0.25	0.00	0.00	0.00	0.00	0.00
61–80 y	0.22	0.00	0.00	0.48	0.00	0.00	0.00	0.00	0.00
>80 y	0.00	0.00	0.00	0.00	0.00	0.00	0.00	0.00	0.00

**Table 4:** Univariate logistic regression analysis to estimate the odds of myocarditis/pericarditis following COVID-19 vaccination.

Parameter	Control (*n* = 668) Number (%)	Case (*n* = 167) Number (%)	Odds Ratio	*p*-Value	95% CI
Sex *					
Male	329 (49.25)	118 (70.66)	2.48	<0.001	1.72–3.58
Female	339 (50.75)	49 (29.34)	reference		
Age group *					
12–17 y	15 (2.25)	136 (81.44)	81.60	<0.001	9.66–689.19
18–20 y	17 (2.54)	9 (5.39)	4.76	0.168	0.52–43.80
21–40 y	251 (37.63)	10 (5.99)	0.36	0.352	0.04–3.11
41–60 y	267 (40.03)	3 (1.80)	0.10	0.057	0.01–1.07
61–80 y	108 (16.19)	8 (4.79)	0.67	0.716	0.08–5.94
>80 y	9 (1.35)	1 (0.60)	reference	-	-
Vaccine type *					
ChAdOx1-nCoV	293 (43.86)	15 (8.98)	reference		
BNT162b2	42 (6.29)	146 (87.43)	67.90	<0.001	36.45–126.49
mRNA-1273	4 (0.60)	3 (1.8)	14.65	0.001	3.01–71.43
BBIBP-CorV	127 (19.01)	0 (0.00)	-	-	-
CoronaVac	202 (30.24)	3 (1.80)	0.29	0.053	0.08–1.02
Vaccine dose					
Dose 1	219 (32.78)	55 (32.93)	reference		
Dose 2	427 (63.92)	109 (65.27)	1.02	0.930	0.71–1.46
Dose 3	22 (3.29)	3 (1.8)	0.54	0.335	0.16–1.88
Period of vaccination ^$^					
March–May	74 (11.08)	1 (0.76)	reference		
June–September	461 (69.01)	14 (10.69)	2.23	0.437	0.29–17.34
October–December	133 (19.91)	116 (88.55)	64.54	<0.001	8.83–471.59
History of prior COVID-19 infection					
No	655 (98.05)	164 (98.20)	reference		
Yes	13 (1.95)	3 (1.8)	0.92	0.900	0.31–3.85

* Variables that were later used in the multivariate analysis. ^$^ The analytical model that included the variable of the time period is shown in Supplementary Table S4.

**Table 5:** Multivariate logistic regression analysis to estimate the odds of myocarditis/pericarditis following COVID-19 vaccination.

Parameter	Adjusted Odds Ratio	*p*-Value	95% CI
Sex			
Male	1.41	0.319	0.72–2.78
Female	Reference		
Age group			
12–17 y	23.89	0.000	7.20–79.30
18–20 y	4.82	0.011	1.42–16.32
21–60 y	0.38	0.056	0.14–1.02
>60 y	Reference		
Vaccine type			
BNT162b2	12.78	0.000	3.05–53.48
mRNA-1273	66.08	0.000	9.81–445.07
ChAdOx1-nCoV	2.26	0.225	0.61–8.40
CoronaVac	Reference		

The independent variables were sex, age group, and vaccine type. The dependent variable was the incidence of myocarditis/pericarditis.

## Data Availability

Not applicable.

## Supplementary Materials

The following are available online at https://www.mdpi.com/article/10.3390/vaccines11040749/s1. Table S1: Characteristics of myocarditis, pericarditis, and myopericarditis following COVID-19 immunization in Thailand. Table S2: Characteristic of myocarditis/pericarditis cases from the review of the medical records. Table S3: Multivariate logistic regression analysis to estimate the odds of confirmed (*n* = 18) and probable (*n* = 26) cases of myocarditis/pericarditis following COVID-19 vaccination. Table S4. Multivariable logistic regression analysis to estimate the odds of myocarditis/pericarditis following COVID-19 vaccine adjusted for period of vaccination.
