# Correction: Mahasing et al. Myocarditis and Pericarditis following COVID-19 Vaccination in Thailand. *Vaccines* 2023, *11*, 749

**DOI:** 10.3390/vaccines11101589

**Published:** 2023-10-12

**Authors:** Chayanit Mahasing, Pawinee Doungngern, Rittichai Jaipong, Poonyaporn Nonmuti, Jirapa Chimmanee, Jurai Wongsawat, Thananya Boonyasirinant, Chaisiri Wanlapakorn, Pattranee Leelapatana, Teerapat Yingchoncharoen, Tachapong Ngarmukos, Kulkanya Chokephaibulkit, Suphot Srimahachota

**Affiliations:** 1 Division of Epidemiology, Department of Disease Control, Ministry of Public Health, Building 10 Floor 3, 88/21 Tiwanon Rd., Nonthaburi 11000, Thailand; pawind@gmail.com (P.D.);; 2 Bamrasnaradura Infectious Diseases Institute, Nonthaburi 11000, Thailand; 3 Faculty of Medicine, Siriraj Hospital, Mahidol University, Bangkok 10700, Thailand; 4 Faculty of Medicine, Chulalongkorn University, Bangkok 10330, Thailand; 5 Faculty of Medicine, Ramathibodi Hospital, Mahidol University, Bangkok 10400, Thailand

The authors wish to make the following corrections to this published paper [1].

In Figure 1, the names of the vaccines have been replaced with their common names, i.e., Comirnaty has been replaced with BNT162B2, AstraZeneca has been replaced with ChA-dOx1-nCoV, Moderna has been replaced with MRNA-1273 and Sinopharm has been replaced with BBIBP-CorV. The corrected Figure 1 is shown below:

In Table 1, we have changed the unit of the number of vaccination doses administered from per 1 million doses to per 100,000 vaccination doses administered and corrected the errors for the overall AEFI rate from 1.95 to be 0.195 per 100,000 doses. We have corrected the number of reported ages for ChAdOx1-nCoV and BNT162b from 21 and 166 to 20 and 165, respectively. The corrected Table 1 is shown below:

In Table 2, we have added a remark about the incidence of myocarditis in ChAdOx1-nCoV under Table 2 and changed the incidence of myocarditis in ChAdOx1-nCoV in children aged 12 to 17 to “NA”. The reason we decided to modify this is to avoid confusion, since ChAdOx1-nCoV was not recommended for people under 18 years old in Thailand, but there were some errors that occurred during the campaign as reported. Therefore, we think it would be clearer for readers if we remove the rate of ChAdOx1-nCoV among vaccinees age 12–17 from the table to the footnote. The corrected Table 2 is shown below:

The authors apologize for any inconvenience this may have caused and affirm that the scientific conclusions remain unaffected. The original publication has also been updated.

## Figures and Tables

**Figure 1:**
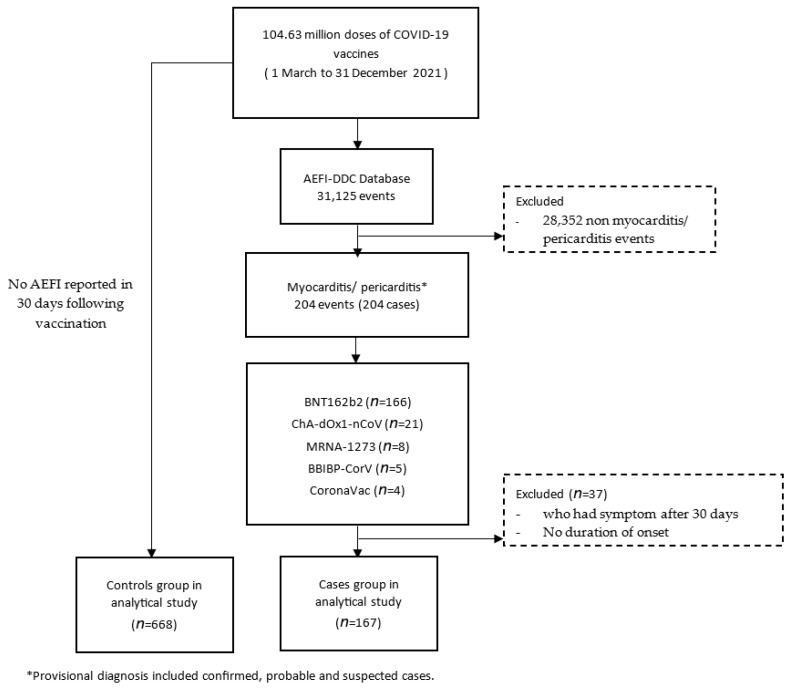
Flow chart of the study.

**Table 1:** Characteristics of the myocarditis/pericarditis cases following immunization in Thailand.

	ChAdOx1-nCoV *n* = 21	BNT162b2 *n* = 166	BBIBP-CorV *n* = 5	CoronaVac *n* = 4	MRNA-1273 *n* = 8	Total *n* = 204
No. of case reports to AEFI-DDC	21	166	5	4	8	204
No. of vaccination doses administered	44,159,927	17,137,233	14,578,943	26,385,393	2,371,390	104,632,886
Rate (cases/100,000 doses)	0.048	0.970	0.034	0.015	0.337	0.195
Reported age, No.	20	165	5	4	8	202
Age, median (IQR), y	61 (41–66)	14 (13–16)	33 (13–47)	23 (21.5–27.5)	39 (32.5–52)	15 (13–17)
Reported onset, No.	20	163	3	3	8	197
Time to symptom onset, median (IQR), d	4 (1–23)	2 (1–4)	5 (0–44)	3 (2–16)	2 (0–6)	2 (1–4)
Reported sex, No.	21	166	5	4	8	204
Male (%)	13 (61.9)	120 (72.29)	2 (40)	2 (50)	3 (37.5)	140 (68.63)
Female (%)	8 (38.1)	46 (27.71)	3 (60)	2 (50)	5 (62.5)	64 (31.37)
Reported occupation No.	21	166	5	4	8	204
Occupation (%)						
Student	3 (14.29)	135 (81.33)	2 (40)	1 (25)	0 (0)	141 (85.45)
HCP	0 (0)	2 (1.2)	0 (0)	0 (0)	0 (0)	2 (1.21)
Monk	0 (0)	2 (1.2)	0 (0)	0 (0)	0 (0)	2 (1.21)
Government officer	0 (0)	1 (0.6)	0 (0)	1 (25)	0 (0)	2 (1.21)
Merchant	1 (4.76)	1 (0.6)	0 (0)	1 (25)	1 (12.5)	4 (2.42)
Unemployed	3 (14.29)	0 (0)	0 (0)	0 (0)	0 (0)	3 (1.82)
Housework	2 (9.52)	0 (0)	0 (0)	0 (0)	0 (0)	2 (1.21)
Employee	4 (19.05)	0 (0)	1 (20)	0 (0)	2 (25)	7 (4.24)
Farmer	1 (4.76)	0 (0)	0 (0)	0 (0)	1 (12.5)	2 (1.21)
Unknown	7 (33.33)	25 (15.06)	2 (40)	1 (25)	4 (50)	39 (19.12)
Reported history of COVID-19 infection, No.	21	166	5	4	8	204
No	20 (95.24)	164 (98.8)	5 (100)	4 (100)	7 (87.5)	200 (98.04)
Yes	1 (4.76)	2 (1.2)	0 (0)	0 (0)	1 (12.5)	4 (1.96)
Reported dose, No.	21	166	5	4	8	204
Dose 1	5 (23.81)	54 (32.53)	3 (60)	4 (100)	2 (25)	68 (33.33)
Dose 2	16 (76.19)	111 (66.87)	2 (40)	0 (0)	2 (25)	129 (63.24)
Dose 3	0 (0)	1 (0.6)	0 (0)	0 (0)	4 (50)	7 (3.43)
Reported prognosis, No.	21	166	5	4	8	204
Status ^$^ (%)						
Full recovery	1 (4.76)	49 (29.52)	1 (20)	0 (0)	1 (12.5)	52 (25.49)
Improved	6 (28.57)	82 (49.4)	0 (0)	1 (25)	4 (50)	93 (45.59)
Death	9 (42.86)	0 (0)	0 (0)	1 (25)	0 (0)	10 (4.90)
Unknown	5 (23.81)	35 (21.08)	4 (80)	2 (50)	3 (37.5)	49 (24.02)
Reported hospitalization, No.	21	166	5	4	8	204
Hospitalization (%)						
IPD	11 (52.38)	139 (83.73)	2 (40)	1 (25)	7 (87.5)	160 (78.43)
OPD	6 (28.57)	18 (10.84)	1 (20)	2 (50)	1 (12.5)	28 (13.73)
ER	2 (9.52)	7 (4.22)	0 (0)	1 (25)	0 (0)	10 (4.90)
Unknown	2 (9.52)	2 (1.2)	2 (40)	0 (0)	0 (0)	6 (2.94)
Reported hospital stay, No.	13	93	2	2	4	114
Duration of hospital stay, median (IQR), in days	3 (1–12)	3 (1–4)	22.5 (10–35)	21 (7–35)	2.5 (1–4.5)	3 (1–5)

IQR: interquartile range; HCP: healthcare provider; ^$^: status was evaluated at date reported. IPD: inpatient department, OPD: outpatient department, ER: emergency room.

**Table 2:** Rate of suspected myocarditis/pericarditis (per 100,000 doses administered) by vaccine, age, and sex (*n =* 202).

Age Group	Vaccine and Sex													
	ChAdOx1-nCoV		BNT162b2		BBIBP-CorV		CoronaVac		MRNA-1273		Total		Background Incidence ^$^ (Cases/100,000 Population)	
	Male	Female	Male	Female	Male	Female	Male	Female	Male	Female	Male	Female	Male	Female
05–11 y	NA	NA	0.00	0.00	0.00	6.21	NA	NA	0.00	0.00	0.00	4.87	0.24	0.29
12–17 y	NA *	NA *	2.87	1.03	2.90	0.00	0.00	0.00	0.00	0.00	2.89	1.02	0.45	0.37
18–20 y	0.14	0.14	1.03	0.47	0.00	0.00	0.00	0.00	0.00	0.00	0.32	0.18	0.87	0.44
21–40 y	0.01	0.01	0.13	0.05	0.00	0.03	0.04	0.04	0.20	0.61	0.03	0.05	0.83	0.51
41–60 y	0.03	0.02	0.07	0.00	0.04	0.04	0.00	0.00	0.57	0.00	0.04	0.02	1.58	1.08
61–80 y	0.19	0.04	0.15	0.00	0.00	0.00	0.00	0.00	0.00	0.61	0.13	0.03	3.88	3.25
>80 y	0.00	0.20	0.00	0.00	0.00	0.00	0.00	0.00	0.00	0.00	0.00	0.11	4.77	4.94

* ChA-dOx1-nCoV was not recommended for use in people less than 18 years old in Thailand; however, some unintentional misuse occurred, and one case of suspected myocarditis was reported among 7826 doses administered to males aged 12–17 years. The only case of suspected myocarditis after the ChAdOx1-nCoV vaccine was a male aged 17 years and 8 months who was reported as 18 years old on the vaccination day and intended to receive the ChAdOx1-nCoV vaccine. His medical record was not available to confirm the diagnosis of myocarditis. ^$^: The background incidence is the 3-year median incidence of myocarditis/pericarditis in 2018–2020 prior to implementation of the COVID-19 vaccine.

## References

[1] Mahasing C., Doungngern P., Jaipong R., Nonmuti P., Chimmanee J., Wongsawat J., Boonyasirinant T., Wanlapakorn C., Leelapatana P., Yingchoncharoen T. (2023). Myocarditis and Pericarditis following COVID-19 Vaccination in Thailand. Vaccines.

