# Supplementary material for: Myocarditis and Pericarditis following COVID-19 Vaccination in Thailand
Source: Vaccines (Basel). 2023 Mar 28;11(4):749. doi: 10.3390/vaccines11040749 (PMC10141407; doi:10.3390/vaccines11040749)
Supplement: Supplementary file 1 [file vaccines-11-00749-s001.zip › vaccines-2201661-supplementary.pdf]

Supplementary Table.

Table S1. Characteristics of myocarditis, pericarditis and myopericarditis following COVID-19 immunization in Thailand (n=199).

	Myocarditis 1 (n=130)	Pericarditis 2 (n=44)	Myopericarditis 3 (n=25)	P-value 1 vs 2	P-value 1 vs 3	P-value 2 vs 3
**Reported age, No.**	129	44	24	0.4138	0.8831	0.8918
Age, median (IQR), y	14 (13-17)	15 (13-17)	15 (13-16)			
**Reported onset, No.**	128	43	25	0.3265	0.6102	0.9287
Time to symptom onset, median (IQR), d	2 (1-4)	2 (1-5)	2 (1-5)			
**Reported gender, No.**	130	44	25	0.011	0.009	0.500
Male (%)	79 (61)	36 (82)	22 (88)			
Female (%)	51 (39)	18 (8)	3 (12)			
**Reported occupation, No**.	101	41	22	0.437	0.731	0.407
Occupation (%)						
student	87 (86)	32 (78)	21 (95)			
HCP	1 (1)	0 (0)	1 (5)			
monk	1 (1)	1 (2)	0 (0)			
Government officer	0 (0)	2 (5)	0 (0)			
merchant	3 (3)	1 (2)	0 (0)			
unemployed	2 (2)	1 (2)	0 (0)			
housework	1 (1)	1 (2)	0 (0)			
employee	4 (4)	3 (7)	0 (0)			
farmer	2 (2)	0 (0)	0 (0)			
**Reported history of COVID-19 infection, No**.	130	44	25	0.309	0.443	-
No	127 (98)	44 (100)	25 (100)			
Yes	3 (2)	0 (0)	0 (0)			
**Reported vaccine type, No.**	130	44	25	0.434	0.266	0.825
ChAdOx1-nCoV	15 (11)	4 (9)	1 (4)			
BNT162b2	104 (80)	38 (87)	22 (88)			
BBIBP-CorV	1 (1)	1 (2)	1 (4)			
CoronaVac	2 (2)	1 (2)	1 (4)			
mRNA-1273	8 (6)	0 (0)	0 (0)			
**Reported dose, No**.	130	44	25	0.331	0.213	0.182
Dose 1	39 (30)	15 (34)	12 (48)			
Dose 2	85 (65)	29 (66)	12 (48)			
Dose 3	6 (5)	0 (0)	1 (4)			
**Report prognosis, No.**	100	34	21	0.394	0.431	0.234
Status$ (%)						
full recovery	33 (33)	9 (26)	10 (47)			
better	59 (59)	24 (71)	10 (47)			
death	8 (8)	1 (3)	1 (5)			
**Report hospitalization, No.**	128	44	25	0.909	0.265	0.296
hospitalization (%)						
IPD	101 (79)	35 (79)	23 (92)			

	Myocarditis 1 (n=130)	Pericarditis 2 (n=44)	Myopericarditis 3 (n=25)	P-value 1 vs 2	P-value 1 vs 3	P-value 2 vs 3
OPD	20 (16)	6 (14)	2 (8)			
ER	7 (5)	3 (7)	0 (0)			
**Reported hospital duration, No.**	59	17	14	0.2838	0.3339	0.9725
hospital duration, median (IQR), d	2 (1-4)	2 (1-3)	2 (1-3)			

Table S2. Characteristic of myocarditis/ pericarditis cases from medical records review.

	ChAdOx1-nCoV	BNT162b2	BBIBP-CorV	CoronaVac	MRNA-1273
**Reported Age (n=71)**	10	57	2	2	0
Median age (IQR), year	56(41-67)	14(13-16)	40(33-47)	23(21-24)	-
**Reported Underlying disease (n=67)**	9	54	2	2	0
No underlying disease	4(44.44)	49(90.74)	2(100)	1(50)	0(0)
Have underlying disease	5(55.56)	5(9.26)	0(0)	1(50)	0(0)
HIV infection	1(20)	1(20)	0(0)	0(0)	0(0)
Diabetes	2(40)	0(0)	0(0)	0(0)	0(0)
Hypertension	3(60)	0(0)	0(0)	1(100)	0(0)
Dyslipidemia	2(40)	0(0)	0(0)	0(0)	0(0)
Gout	1(20)	0(0)	0(0)	0(0)	0(0)
Panic disorder	1(20)	0(0)	0(0)	0(0)	0(0)
G6PD deficiency	0(0)	1(20)	0(0)	0(0)	0(0)
Asthma	1(20)	1(20)	0(0)	0(0)	0(0)
Ischemic heart disease	0(0)	1(20)	0(0)	0(0)	0(0)
chronic pelvic pain	0(0)	1(20)	0(0)	0(0)	0(0)
psychiatric	1(20)	0(0)	0(0)	0(0)	0(0)
thyrotoxicosis	0(0)	0(0)	0(0)	1(100)	0(0)
**Previous AEFI history (n=51)**	5	45	0	1	0
No	5(100)	44(97.78)	0(0)	1(100)	0(0)
Yes	0(0)	1(2.22)	0(0)	0(0)	0(0)
**History drug/ vaccine allergy (n=59)**	8	50	0	1	0
No	8(100)	45(90)	0(0)	1(100)	0(0)
Yes	0(0)	5(10)	0(0)	0(0)	0(0)
**Symptoms (n=71)**	10	57	2	2	0
chest pain	6(60)	56(98.25)	2(100)	1(50)	0
Fever	3(30)	22(38.6)	0(0)	0(0)	0
tachycardia	3(30)	21(36.84)	0(0)	0(0)	0
Dyspnea	4(40)	18(31.58)	0(0)	2(100)	0
Palpitation	3(30)	12(21.05)	0(0)	1(50)	0
vomiting	1(10)	4(7.02)	0(0)	0(0)	0
headache	1(10)	4(7.02)	0(0)	0(0)	0
syncope	3(30)	2(3.51)	0(0)	0(0)	0
lethargy	1(10)	2(3.51)	0(0)	0(0)	0
seizure	2(20)	2(3.51)	0(0)	1(50)	0
diarrhea	0(0)	2(3.51)	0(0)	0(0)	0
sweating	1(10)	1(1.75)	0(0)	0(0)	0
**Abnormal finding**					
**Troponin level (n=49)**	5	41	2	1	0
Elevated	3(60)	25(60.98)	2(100)	1(100)	31(63.27)

normal	2(40)	16(39.02)	0(0)	0(0)	18(36.73)
**Electrocardiogram (EKG) (n=54)**	7	44	2	1	0
new ST-elevation/ PR-depression	2(28.57)	16(36.36)	1(50)	0(0)	0(0)
other abnormal	3(42.86)	11(25)	0(0)	1(100)	0(0)
normal	2(28.57)	17(38.64)	1(50)	0(0)	0(0)
**Echocardiogram (n=23)**	3	17	2	1	0
Abnormal cardiac function	2(66.67)	3(17.65)	0(0)	0(0)	0(0)
Normal	1(33.33)	14(82.35)	2(100)	1(100)	0(0)
**Magnetic resonance imaging (n=5)**	1	3	1	0	0
abnormal	1(100)	3(100)	1(100)	0(0)	0(0)
normal	0(0)	0(0)	0(0)	0(0)	0(0)

Table S3. Multivariable logistic regression analysis to estimate the odds of confirmed (n=18) and probable (n=26) cases of myocarditis/ pericarditis following COVID-19 vaccine.

parameter	Adjusted Odds ratio	P-value	95% CI
**Gender**			
Male	1.99	0.186	0.72-5.53
Female	reference		
**age group**			
12-17 y	26.77	0.001	3.84-186.65
18-60 y	1.07	0.937	0.20-5.68
>60 y	reference		
**vaccine type**			
BNT162b2	18.72	0.015	1.77-198.16
MRNA-1273*	-	-	-
ChAdOx1-nCoV	3.23	0.296	0.36-29.18
BBIBP-CorV*	-	-	-
CoronaVac	reference		

*No confirmed/ probable case reported on MRNA-1273 and BBIBP-CorV.

Table S4. Multivariable logistic regression analysis to estimate the odds of myocarditis/ pericarditis following COVID-19 vaccine adjusted for period of vaccination.

Parameter	Adjusted Odds ratio	P-value	95% CI
**Gender**			
Male	1.60	0.200	0.78-3.30
Female	reference		
**Age group**			
12-17 y	15.22	<0.001	4.01-57.80
18-20 y	3.27	0.085	0.85-12.60
21-60 y	0.29	0.021	0.10-0.83
>60 y	reference		
**Vaccine type**			
BNT162b2	6.53	0.076	0.82-52.10
mRNA-1273	34.48	0.004	3.02-394.20
ChAdOx1-nCoV	2.38	0.361	0.37-15.24
CoronaVac	reference		
**Period of vaccination***			
Mar-May	reference		
Jun-Sep	0.57	0.657	0.05-6.64
Oct-Dec	2.15	0.559	0.17-27.77

* During the 10 months of the COVID-19 vaccination campaign, we have divided into 3 time periods according to the vaccine introduction including March – May, June – September, and October – December.
